# Hemolysis Derived Products Toxicity and Endothelium: Model of the Second Hit

**DOI:** 10.3390/toxins11110660

**Published:** 2019-11-13

**Authors:** Marie Frimat, Idris Boudhabhay, Lubka T. Roumenina

**Affiliations:** 1 Univ. Lille, U995-LIRIC-Lille Inflammation Research International Center, F-59000 Lille, France; 2 CHU Lille, Department of Nephrology, F-59000 Lille, France; 3 Centre de Recherche des Cordeliers, INSERM, Sorbonne Université, Université de Paris, F-75006 Paris, France; idris.boudhabhay@gmail.com

**Keywords:** damage-associated molecular patterns (DAMPs), endothelial cells, glomerular endothelium, heme, hemolytic diseases, two-stage model disease

## Abstract

Vascular diseases are multifactorial, often requiring multiple challenges, or ‘hits’, for their initiation. Intra-vascular hemolysis illustrates well the multiple-hit theory where a first event lyses red blood cells, releasing hemolysis-derived products, in particular cell-free heme which is highly toxic for the endothelium. Physiologically, hemolysis derived-products are rapidly neutralized by numerous defense systems, including haptoglobin and hemopexin which scavenge hemoglobin and heme, respectively. Likewise, cellular defense mechanisms are involved, including heme-oxygenase 1 upregulation which metabolizes heme. However, in cases of intra-vascular hemolysis, those systems are overwhelmed. Heme exerts toxic effects by acting as a damage-associated molecular pattern and promoting, together with hemoglobin, nitric oxide scavenging and ROS production. In addition, it activates the complement and the coagulation systems. Together, these processes lead to endothelial cell injury which triggers pro-thrombotic and pro-inflammatory phenotypes. Moreover, among endothelial cells, glomerular ones display a particular susceptibility explained by a weaker capacity to counteract hemolysis injury. In this review, we illustrate the ‘multiple-hit’ theory through the example of intra-vascular hemolysis, with a particular focus on cell-free heme, and we advance hypotheses explaining the glomerular susceptibility observed in hemolytic diseases. Finally, we describe therapeutic options for reducing endothelial injury in hemolytic diseases.

## 1. Introduction 

The endothelium, a monolayer of endothelial cells (ECs) covering the entire vascular tree, is a huge exchange surface located at the interface between the vascular and tissue sectors. Acting as a key player in many vascular functions (e.g., vasomotor tone, permeability, immunity, coagulation, angiogenesis), it is both a crucial actor in homeostasis and at the forefront of many pathological processes [1]. 

Endothelium is traditionally described under two states. Under physiological conditions, the healthy endothelium is considered as quiescent and the normal average life span of ECs is more than one year [2]. In this context, endothelium permeability is limited and it serves the physiological functions of nutrient transfer. Quiescent endothelium preferentially synthesizes vascular relaxation factors and prevents the formation of thrombi due to the non-thrombogenic, non-coagulant, pro-fibrinolytic properties of its surface. It expresses few leukocyte adhesion molecules. Nevertheless, in response to a wide variety of environmental, chemical, physical, or humoral stimuli, ECs may become activated [3]. If the stimuli are too strong and exceed the adaptation capacity of the EC, toxic effects are observed leading to acquisition of a proinflammatory and prothrombotic profile, cell damage, or even death. These toxic effects have severe consequences for the organism, resulting in different pathological conditions. 

To apprehend the endothelium as a two-state ‘on/off’ organ is simplistic, however. The ‘on’, or activated, state is not unique but multiple and dynamic, and may progress to a pathological state. Various factors determine the endothelial response to a stimulus, starting with the characteristics of the endothelium itself. A vast heterogeneity of structure and function exist according to vascular segments (arteries, arterioles, capillaries, post-capillary veins, veins) and perfused tissues as illustrated by different gene expression profiles [4]. This could partly explain the specific location of expression in some vascular diseases, such as thrombotic microangiopathies (TMA) being limited to small vessels [5]. ECs’ features also change over time, according to age (e.g., higher susceptibility to thrombosis and oxidative stress in elderly people [6,7]) or the hormonal status (e.g., glomerular endotheliosis reported during some pregnancies [8]). In addition to these intrinsic determinants, the activated state of the endothelium can be modulated by the characteristics of the stimulus (nature, intensity), but also by the competences of various systems involved in the regulation of vascular homeostasis. For example, having a weaker complement regulation system due to factor H or membrane cofactor protein (MCP) pathogenic variants predisposes to atypical hemolytic uremic syndrome (HUS), but is insufficient to trigger the disease itself. Other events/factors are necessary to initiate or sustain the activation of endothelium. Therefore, we consider endothelial activation is not merely ‘on’ or ‘off’ but is rather a continuous, multistate process that becomes pathological when regulation/repair systems are overwhelmed through overstimulation (multiple hits) and/or underperforming regulation systems. 

Here we will illustrate this concept using the example of intravascular hemolysis. Evidence suggests that in hemolytic diseases, after an initial stimulus which activates endothelium and/or causes RBC lysis, the cell-free hemoglobin and heme act as a second ‘hit’, leading to intolerable endothelial stress and resulting in tissue and organ injury. Hemoglobin (Hb) is the oxygen carrier protein in the red blood cells (RBC) and heme is its oxygen-binding prosthetic group. Hb and heme do not circulate freely under physiological condition—a prior pathological event is required for their release—and free Hb and heme constitute powerful endothelial aggressors through both overstimulation and saturate the regulatory systems [9]. Therefore, if not efficiently eliminated, Hb and heme trigger endothelial activation and damage. 

The present review summarizes current knowledge of the complex process of endothelial activation by Hb and heme with a specific focus on renal endothelium and its potential susceptibility to this ‘toxin’. 

## 2. Hemolytic Diseases

### 2.1. Hemolytic Diseases and Intravascular Hemolysis 

Hemolysis is defined by a shortening of the ~120 days life span of the circulating RBC [10]. This pathological event can occur in a large spectrum of diseases summarized in Table 1. The specific impact of a hemolytic episode differs according to various parameters, among which the extra- or intravascular location of RBC lysis exert a major influence. Extravascular hemolysis is more particularly due to defects in RBC, which are responsible for reduced deformability and favors sequestration by the spleen and/or a higher recognition by systems involved in RBC destruction such as the complement, antibodies, and phagocytic cells (especially macrophages). These different mechanisms drive a premature removal of RBC in the spleen, and also in the blood marrow and liver [11,12,13]. Iron overload and its consequences are prominent in the clinical presentation of extravascular hemolytic diseases, though direct effects upon the endothelium are few. On the other hand, the endothelium is a key target for intravascular hemolysis through the release of powerful proinflammatory mediators. 

In many cases, an intravascular hemolysis event is an additional complication during the progression of an extravascular hemolytic disease. Acute vascular effects mediated by intravascular hemolysis add to the chronic complications of extravascular hemolytic disease. Typically, in sickle cell disease (SCD), the shortened lifespan of sickle RBCs includes both extravascular mechanisms of removal (primarily through the reticuloendothelial system) and intravascular hemolysis which directly contributes to the vaso-occlusive process, the most acute complication of SCD [10,17]. Other arguments support a link between the intravascular topography of hemolysis and direct endothelial suffering. For example, splenectomy treatment in b-thalassemia patients was related with significantly increased intravascular hemolysis, in association with elevations of inflammation markers (soluble E-selectin, P-selectin, hs-CRP) and hypercoagulability (activation of thrombin-antithrombin complexes) [18]. Conversely, the intravascular component inhibition of a hemolytic disease is associated with a decrease of thrombotic complications. This is well-known in paroxysmal nocturnal hemoglobinuria (PNH) in which the eculizumab treatment—a human monoclonal antibody against the C5 component of complement that blocks the terminal complement pathway—facilitates abrogate intravascular hemolysis. In parallel, the risk of thrombosis drastically decreased in treated patients (relative reduction, 85%; absolute reduction, 6.3 TE events/100 patient-years) [19]. Interestingly, eculizumab treatment in PNH has also been associated with de novo extravascular hemolysis. PNH RBCs are protected from hemolysis because of the inhibition of C5, but C3-opsonized RBCs may be removed by macrophages, thereby favoring extravascular hemolysis [20]. Thus, the switch from intravascular to extravascular hemolysis modulates the phenotype of patients who have no further thrombotic complications, but who may develop iron overload [20,21]. 

Endothelial suffering in intravascular hemolysis is directly related to the release of large quantities of RBC damage associated molecular patterns (DAMPs) and other mediators close to ECs. Heat shock protein (Hsp) 70 could stimulate monocytes/macrophages via the TLR2, TLR4, and CD14 pathways [22]; adenosine 5’ triphosphate (ATP) is rapidly degraded to adenosine 5’ diphosphate (ADP) [10,23], and the increase of ADP levels have been associated with promotion of sickling, hemolysis, and damage to multiple tissues in a SCD model [24]; RBC microvesicles can transfer heme to ECs and trigger endothelial activation [25]. In addition, free heme issued from released Hb has a major impact on endothelium and this review will focus more particularly on this powerful proinflammatory mediator, the most-studied of the products derived from hemolysis. 

### 2.2. Concentration of Free Heme during Intravascular Hemolysis

Despite clear evidence that cell-free heme is released into the circulation after RBC breakdown, its precise concentration and how exactly it exerts its harmful effects remain unclear. The majority of the available methods for heme quantification in fact measure the total protein-bound heme, which does not distinguish between cell-free heme, heme-albumin, heme-hemopexin (Hx), and Hb. Such colorimetric tests are commercially available but their results must be interpreted and reported with care, since they do not permit evaluation of the amount of free heme. The heme-scavenging capacity of plasma is enormous and even if Hx is depleted, multiple proteins can bind heme with low affinity [26]. The use of colorimetric methods resulted in the notion that free heme can reach concentrations of up to 25–50 µM in plasma of patients [27,28] or 75–100 µM in mice with SCD [29], which is likely largely overestimated. Another method, based on the recognition of heme by an antibody, suggests that only 2–5µM heme are protein-free during experimental intravascular hemolysis [30]. Spectroscopic approaches are also proposed but give contradictory results of, on average, 20µM (several patients up to over 50µM) [31] to only a few µM for patients with SCD [32,33]. Further studies are needed to precisely define the amount of cell-free heme outside Hb as well as the levels of protein-free heme. Moreover, it is important to distinguish the concentration of cell- and protein-free heme in the circulation, where it will be in immediate contact with the heme-scavenging protein, versus the amount of heme in the microcirculation in the context of TMA or SCD vaso-occlusion. In these latter cases, large amounts of RBC will breakdown in a confined space with a perturbed blood flow, resulting in a much higher local concentration of heme compared with the circulation. Further studies are needed to develop a methodological approach permitting accurate estimation of the local heme concentration.

### 2.3. Others Markers of Intravascular Hemolysis 

Thus, there is no method yet available for determining serum heme concentrations during intravascular hemolysis and other clues are therefore used in clinical practice. Reduced RBC counts and Hb rates are nonspecific, but they are important indicators of severity; hemoglobin values at diagnosis have thus been correlated with the risk of death and the requirement for multiple therapies in auto-immune hemolytic anemia (AIHA) [34]. A potential indicator of an intravascular process could be the kinetics of erythrocyte lysis: in intravascular hemolysis, the RBC breakdown rate was calculated as being 200 mL RBC per hour, whereas it was 10-fold less in extravascular hemolysis [16]. Also, increased bilirubin levels due to the rise of Hb catabolism (mainly resulting in unconjugated hyperbilirubinemia) and reticulocytosis, an indicator of marrow compensating for RBC loss, are not sufficiently specific to discriminate intra- from extra-vascular hemolysis. Reticulocytosis could be more intensive in acute hemolytic crisis, more generally the prerogative of intravascular events; however, various factors influence this parameter which may be inadequate (e.g., marrow involvement, iron/vitamin deficiency) or absent in subclinical hemolysis with normal or slightly decreased hemoglobin levels, as observed in patients with prosthetic valve replacement, for example [35]. Plasma haptoglobin depletion is mainly attributed to the direct release of free Hb into the circulation during intravascular hemolysis. However, this does not exclude the classical extravascular causes because some structurally-altered RBC may escape reticuloendothelial clearance and be destroyed in the circulation. In a study in 479 patients, this parameter was indeed not discriminant between intra- or extravascular etiologies [36]. More specific for intravascular hemolytic events is the increase of plasma lactate dehydrogenase (LDH), particularly isoenzymes 1 and 2, which are strongly expressed in RBC and thus liberated into circulation during lysis. In addition, the increase/decrease of LDH is generally an early sign of response to treatment/relapse, respectively. Hemosiderinuria, which reflects the presence of hemosiderin bound to iron in the urine (typical ‘brownish’ in color), is also a good indicator of intravascular hemolysis and highlights the major role of the kidney in the clearance of iron. Finally, schizocytes indicate intravascular hemolysis issued from mechanical fragmentation of RBC due to an obstacle within the vessels. This points to diagnosis of TMA with a count generally >2% (normal <0.5%). A moderate increase of the schizocyte level may also occur in cases of disseminated intravascular coagulation or intravascular devices (e.g., mechanical artificial hearts, valves).

## 3. Heme—Its Structure, Metabolism, and Detoxification 

### 3.1. Heme: Structure and Nomenclature

Heme is an ubiquitous, iron-containing compound found in large amounts in many cells and organisms that serves, through different hemoproteins, fundamental cellular processes such as electron transfer of the respiratory chain or oxidase and peroxidase enzyme reactions [37]. The heme prosthetic group forms a tetrapyrrole ring—also called protoporphyrin IX—with an iron atom in its center. This central iron had six ligand binding sites, four of which are occupied by nitrogen atoms of the tetrapyrrole ring; the two remaining binding sites can be occupied by gases—such as oxygen, nitric oxide (NO), and carbon monoxide (CO)—or by amino acids [38].

**Figure 1:**
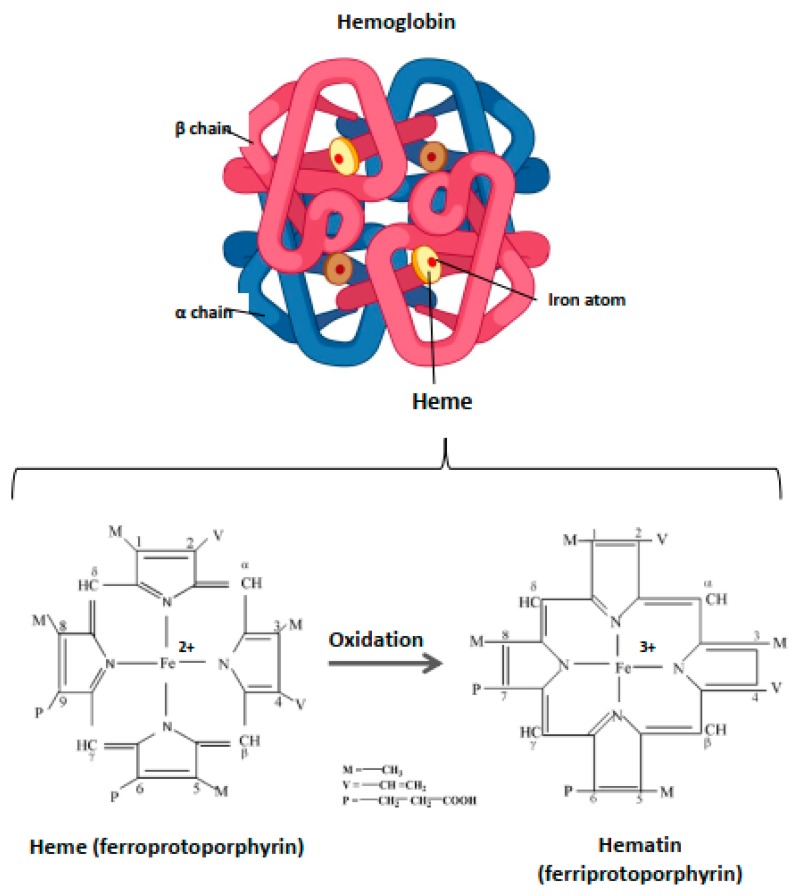
Structure of hemoglobin and heme. Hb is a hemoprotein belonging to the globulin superfamily. It is a 64 kDa heterotetramer, formed by four globular subunits—globins—and four heme molecules. Globins constitute the protein part of Hb. Each Hb molecule consists of two pairs of polypeptide chains in adults, α and β (α2β2). Each globin carries in its center a heme molecule, which is formed by a protoporphyrin IX and, in basal condition, a ferrous iron atom (Fe^2+^). Heme is located in a hydrophobic ‘pocket’, an environment that protects the iron atom from oxidation and allows it to be maintained in ferrous state (Fe^2+^) during the oxygenation/deoxygenation stages. In the event of oxidation, this atom loses an electron, thus passing to the ferric state (Fe^3+^). The heme is then transformed to hematin and the hemoprotein, methemoglobin, or ferrihemoglobin.

Within the blood vessels, this small molecule of ~620 Da is strictly compartmentalized into the RBC where it is an essential component of Hb, enabling its function as a gas carrier. Hb includes four heme prosthetic groups, each located in a globin chain (Figure 1). The iron atom is the focal point of Hb due to its catalytic properties and the capacity to assure the delivery of molecular oxygen delivery to tissues. In the case of oxidation processes, the ferrous iron (Fe^2+^) loses an electron, thus passing to the ferric state (Fe^3+^). Heme is then called hematin, and hemoglobin (OxyHb(Fe^2+^)) is called ferrihemoglobin or methemoglobin (MetHb(Fe^3+^)) (Figure 1). Within Hb, heme is contained in a hydrophobic pocket to maintain its iron in a Fe^2+^ state, required for oxygenation/deoxygenation steps, and to limit oxidation processes [39]. Owing to its potent pro-oxidative potential, the catabolism of heme from senescent erythrocytes is finely regulated. 

### 3.2. Heme Catabolism: The Key Role of Heme-Oxygenase

Under normal, healthy conditions there is no direct interaction between endothelial cells and heme and RBC catabolism mainly occurs in the extravascular compartment. Following an average lifespan of 120 days (earlier in some pathological conditions), mature human RBC become senescent; they are then assimilated by macrophages of the reticuloendothelial system (RES) in bone marrow and spleen, more anecdotally in liver, via a process called erythrophagocytosis [40,41]. Approximately 270 billion hemoglobin molecules in each RBC, and roughly 2 million senescent RBC, are thus recycled every second [41]. The RBC-containing phagosome merges with lysosomal vesicles where red cells are degraded. Heme is transported from the phagolysosome to the cytosol by a heme-transporting permease called HRG1 (SLC48A1) [42], where it can be metabolized in a rate-limiting step by heme-oxygenase (HO). Interestingly, HRG1-deficient mice accumulate high concentrations of heme in the lysosomes of macrophages by crystallizing them into hemozoin, which heretofore has only been found in blood-feeding organisms [43]. Hemozoin could therefore represent a previously unsuspected heme tolerance pathway in mammals.

**Figure 2:**
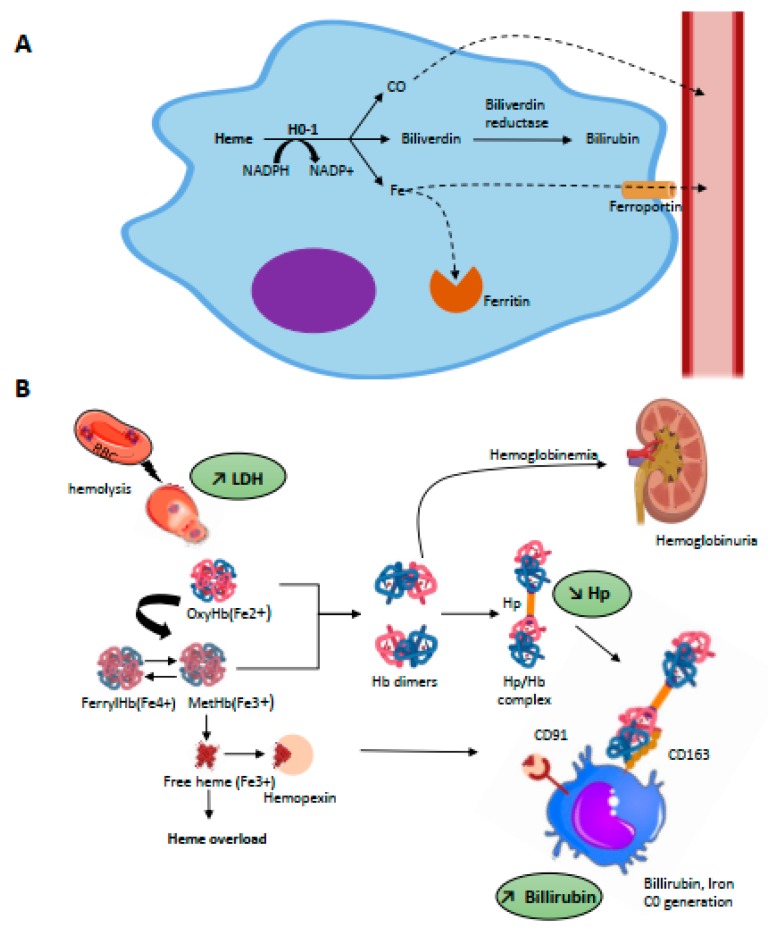
(**A**) Intracellular metabolism of heme. Heme metabolism is driven by heme-oxygenases (HO-1, inducible and HO-2, constitutive) through a NADPH-dependant addition of an oxygen molecule to the porphyrin ring of heme which catalyses the oxidation of heme and release equimolar quantities of iron, biliverdin, and carbon monoxide (CO). Intra-cytosolic iron will be either stored by ferritin or exported in the plasma through the iron transporter, ferroportin. The billiverdin reductase will reduce billiverdin into billirubin. CO product binds to circulating hemoglobin and is transported as carboxyhemoglobin until it is excreted through ventilation. (**B**) Scavenging of hemolysis-derived products. Intravascular hemolysis induces release of RBC content including OxyHb(Fe2+) and MetHb(Fe3+). Free Hb is scavenged by hp and the complex captured by CD163 expressed on mature tissue macrophages, promoting its endocytosis. Globin degradation then occurs in lysosomes and the majority of heme is metabolized by HO-1 in cytosol. Hp is rapidly consumed, and free Hb dimers may be filtered into the kidneys, leading to hemoglobinuria. Overload of MetHb leads to the release of free heme, which can be scavenged by Hx. The complex is recognized by CD91 expressed on macrophages. Hb, then heme degradation generate bilirubin, biliverdin, iron, and CO.

HO is the main heme catabolism enzyme; it drives the NADPH-dependent addition of an oxygen molecule to the porphyrin ring of heme, thus catalyzing the oxidation of heme and the equimolar release of biliverdin, free iron, and CO [44] (Figure 2A). Iron is then either stored in ferritin or is exported for re-use from macrophages to plasma via the transmembrane ferrous exporter ferroportin [45]; biliverdin is enzymatically converted to bilirubin via biliverdin reductase which, similar to CO, has powerful anti-oxidant properties [46]. 

Two isoforms of HO are known: HO-1—an inducible isoform and HO-2—a constitutive isoform [47]. They are anchored in the membrane of endoplasmic reticulum with a cytosolic orientation [48] and both have a catalytic activity towards heme. HO-2 is constitutively expressed, especially in the central nervous system where its neuroprotective properties have been demonstrated [49]. HO-2 could also play a role in vascular homeostasis: the deletion of *HO-2* has been associated with oxidative stress, inflammation, and angiogenesis both in vivo [50] and in vitro in ECs [51]. HO-1 is better documented: its basal expression is weak in normal tissues, except in those involved in the removal of senescent erythrocytes such as in the liver and spleen, thereby highlighting its crucial role in erythrophagocytosis [37]. It is transcriptionally upregulated by various stimuli such as oxidative stress, inflammatory cytokines, or iron-containing molecules. Heme itself is a strong inducer of HO-1 expression through its binding to the transcriptional repressor BACH1, leading to its proteasomal degradation. NFR2, a major regulator of the anti-oxidant stress response, can thus bind to HO-1 promotor and induce transcription [52,53]. Hence, by degrading heme, generating powerful anti-oxidant compounds (CO and bilirubin), but also stimulating ferritin production which binds the iron, HO-1 is considered to offer significant defense against oxidative stress [54]. Deficiency of HO-1 is thus associated with persistent hemolytic anemia, iron accumulation in tissues, chronic inflammation, and microcirculation disturbances in both humans [55,56] and mice [57]. Conversely, overexpression of HO-1 contributes to the resolution of inflammation and vascular dysfunction, suggesting the upregulation of HO-1 as a therapeutic strategy for various diseases, especially cardiovascular [58,59] and renal diseases [60]: this strategy remains controversial, however [61]. 

Moderate intravascular hemolysis is a common condition in newborns and is followed by the accumulation of heme-derived bilirubin, which is a secondary product of the activity of HO-1. Although liver macrophages are a major site of enzymatic heme breakdown in adults, proximal tubules in the kidneys could perform the functions of both heme uptake and catabolism in mouse neonates [62]. Thanks to the activity of HO-1, neonatal jaundice is a benign process that is resolved by the end of the first week of life without treatment.

It should be noted that a small proportion of heme may also be effluxed from the cell by the membranal heme exporter, FLVCR1a [63]. The loss of endothelial *FLVCR1a* in in vitro and in vivo models has thus been associated with an accumulation of intracellular heme responsible for increased cell death by paraptosis [64]. Even in cases of massive hemolysis, the rate of circulating heme should be relatively low in circulation. This is supported by biophysical evaluation of the Hx-binding capacity of heme in different states [65]. Indeed, in NaOH-dissolved hemin (used in the majority of the studies as a source of heme), approximately 80% is available for Hx binding, while this was only 10% in a pre-formed, heme-albumin complex. These observations suggest that in any physiological scenario in which heme might be present in extracellular spaces as a component of a natural hemoprotein, the concentration of free or quasi-free heme can be expected to be very low. Extracellular heme binds plasma exporters, especially Hx detailed below, which transfer it into other cells [37].

### 3.3. Defense Mechanisms against the Toxicity of Hemolysis-Derived Products 

#### 3.3.1. Scavengers of Circulating Free Hb and Heme 

To counteract the toxicity of Hb and derived products, mammalians possess specific protective mechanisms, in particular the serum proteins haptoglobin (Hp) and hemopexin (Hx) (Figure 2B). 

Hp is an abundant, plasmatic glycoprotein with normal range concentrations of 0.5–3 g/L, which corresponds to a Hb binding capacity of 0.3–1.8 g/L [66]. Belonging to acute inflammation proteins, its plasmatic level increases in the presence of pro-inflammatory cytokines; conversely, this drops to virtually zero in cases of intravascular hemolysis due to receptor-mediated removal of Hp in complex with Hb. Indeed, Hp shares extensive interactions with different sub-units of dimeric Hb, explaining the very high-affinity interaction between these proteins with a dissociation constant (Kd) reported to be as low as 10^−12^–10^−15^ M [67,68]. This binding prevents oxidative damage in cells and tissues, although radicals are still formed within the Hb-Hp complex [69,70]. Hp could serve as a restrictor of radical migration within Hb [71]. Furthermore, Hp may stabilize the region of the globin moiety, which in turn may prevent heme release [71]. Hb-Hp complexes are recognized by the cluster of differentiation receptor 163 (CD163) [72], which is exclusively expressed by cells of the monocyte macrophage lineage and has particularly high expression levels in red pulp macrophages of the spleen and Kupffer cells of the liver [73]. This recognition promotes the complex’s endocytosis by macrophages, thus leading to globin degradation in lysosomes and heme catabolism by HO-1 in cytosol, as detailed above. There is an exponential relationship between Hp availability relative to oxyHb(Fe^2+^) and resultant protective activity. In vitro, at least iso-stoichiometric quantities of Hp relative to oxyHb(Fe^2+^) are necessary to provide endothelial protection against lipid peroxidation, explaining the large quantities of Hp required to prevent Hb toxicity [74]. Once the binding capacities of Hp are exceeded, Hb oxidation can occur and leads to ferrihemoglobin and thence to the release of free heme. 

Free heme is quickly scavenged by a second plasmatic glycoprotein involved in the detoxification process: hemopexin (Hx) [75]. Similar to Hp, the expression of Hx is inducible under inflammatory conditions [76] and its plasmatic rates drop during intravascular hemolysis events [27]; indeed, the majority of both of these two proteins are not reclaimed after endocytosis but degraded in lysosomes [77,78]. The affinity of the heme-Hx complex is also very high with a KD < 10^−13^ M, which makes it virtually irreversible [73]. Upon heme-Hx complex formation, Hx undergoes a conformational change allowing it to bind to CD91, a multi-ligand scavenger receptor that belongs to the LDL receptor superfamily [77]. In vivo, the heme-Hx complex is primarily cleared by hepatocytes via CD91-mediated endocytosis [29]—but this receptor is also expressed by others cells, especially macrophages. In cells heme is managed by HO-1, as detailed above, and its iron reclaimed. By scavenging free heme, Hx prevents heme-mediated oxidative reactions, activation of immune receptors and vascular inflammatory processes [79]. Sub-stoichiometric quantities of Hx are sufficient to significantly attenuate Hb-triggered lipid peroxidation and endothelial damage [74]. Conversely, heme-overloaded Hx-null mice show increased oxidative stress in blood vessels and endothelial activation [29]. Other proteins are able to bind heme, though with lower affinity, such as albumin (Kd ≈ 10^−8^ M), lipocalin α1 microglobulin or lipoproteins (Kd ≈ 10^−10^ M). However, the binding of albumin to heme is reversible and can transfer heme to Hx [80]. Moreover, none of these proteins can efficiently and fully block the pro-oxidant and proinflammatory effects of heme, nor prevent its intercalation into lipid membranes, though this translocation is completely blocked in the presence of hemopexin [73,81].

#### 3.3.2. Cellular Adaptation Mechanisms to Heme Overload

In response to heme-mediated oxidative stress, eukaryotic cells activate the transcription factor NRF2. Among its target genes is not only HO-1 (described in detail above), but also other mediators of antioxidant responses such as p62/SQTM1 sequestosome-1 [82]. In response to heme, p62/SQTM1 aggregates are formed both in vitro and in hemolysis models in vivo. They contain ubiquitinated proteins in structures known as aggresome-like induced structures (ALIS) and are an adaptation mechanism allowing cells to cope with the excess heme. This type of response is demonstrated in macrophages in vitro and in total kidney, spleen, and liver tissue, but the induction of ALIS by heme in ECs has not been investigated. 

## 4. Harmful Effects of Heme on Endothelium 

**Figure 3:**
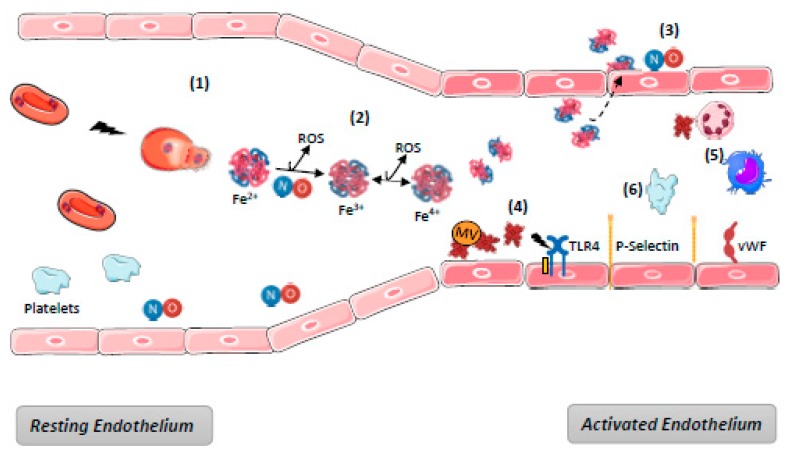
Harmful effects of hemolysis. (**1**) Hemolysis induces release of RBC content including OxyHb; (**2**) Ferric (Fe3+)MethHb results from OxyHb oxidation either spontaneous or through NO binding, generating its depletion and producing reactive species of oxygen (ROS). Oxidation of (Fe3+)MethHb in (Fe4+)FerrylHb increases the formation of ROS. (**3**) dimers of Hb diffuse through endothelium barrier and deplete NO; (**4**) RBC microvesicles (MV) facilitate heme transfer to ECs where it mediates endothelial injury, TLR4 activation, P-selectin, and vWF exocytosis from WPB. Overall, the endothelium become a pro-inflammatory and pro-thrombotic surface; (**5**) Platelet activation promoted by NO depletion increases the pro-thrombotic state; (**6**) Free heme promotes recruitment and activation of neutrophils and macrophages activation through TLR4. NB: complement and hemostasis systems activation are not represented—see Figure 4.

In cases of massive hemolysis, scavenging systems are overwhelmed, leading to the accumulation of free Hb and heme in blood. Endothelium, although it is not the only target [9,83,84], appears to be in the front line with respect to hemolysis-derived products. Their mechanisms of toxicity are generally described as time- and concentration-dependent. They are various, either directly due to the biochemical properties of free Hb and heme, or else indirect as a consequence of the interactions between heme and the actors in endothelium defense mechanisms. The main mechanisms of hemolysis-mediated toxicity for endothelium are detailed below and summarized in Figure 3.

### 4.1. Direct Heme-Mediated Toxicity 

It should be noted that a part of plasma heme may bind to membrane vesicle structures generated from erythrocytes breakdown. RBC microvesicles might then transfer heme to EC and induce endothelial damage [25]. The remaining free Hb and heme are bioavailable for various biochemical reactions

#### 4.1.1. Redox Reactions and ROS Generation

Free Hb undergoes oxidation via different mechanisms: (1) Ferrous iron (Fe^2+^) included into oxyHb can spontaneously auto-oxidize in plasma, generating ferric protein MetHb(Fe^3+^) and the superoxide radical (O_2_^−^). Superoxide radical anions can then dismutate to hydrogen peroxide (H_2_O_2_), and the ferric protein can react with this peroxide to induce the formation of a reactive ferryl iron (Fe^4+^) and a free radical bound to the protein (R^+^); (2) having a very high affinity for nitric oxide (NO), oxyHb(Fe^2+^) induces an irreversible NO dioxygenation reaction producing MetHb(Fe^3+^) and nitrate (NO3^-^). Moreover, MetHb(Fe^3+^) can release free heme, which in turn release Fe^3+^ once Hp is exceeded. 

All these molecules—free iron and radicals—are highly reactive, oxidizing, and able to interact with lipids, proteins, DNA and other molecular cell or tissue components. These unwanted modifications can be responsible for damaging and disrupting cell membranes [85,86]. 

#### 4.1.2. Lipophilic Properties 

Heme is a very hydrophobic molecule that is only stable within the heme pocket of a heme protein and cannot persist as a monomeric, soluble molecule in blood. Owing to its lipophilic nature, heme can interleave within lipid-rich cell membranes, thus increasing their susceptibility to damage via oxidation mechanisms. For example, it has been shown that heme rapidly intercalates into the plasma membrane of cultured ECs and can serve as a source of highly-damaging Fe [87]. Heme can also affect the erythrocyte membranes and contribute to the amplification of hemolysis. Indeed, it can alter the conformation of erythrocyte cytoskeleton proteins, such as spectrin and protein 4.1, thereby reducing their structural stability [88,89], especially in the case of senescent erythrocytes. 

Another consequence is a high affinity for lipoproteins, particularly low-density lipoprotein (LDL). Indeed, heme can spontaneously insert itself into LDL particles and promote their oxidative modification, processes which can be amplified by H_2_O_2_ [90]. In addition to LDL oxidation, this results in the generation of lipid peroxidation products and heme degradation (by oxidative scission of the porphyrin ring) and so iron release that, in turn, can amplify oxidative processes [91]. Heme-induced LDL oxidation contributes to induce vascular damage, as suggested by the direct cytotoxicity of oxidized LDL to ECs. Accordingly, hemopexin in stoichiometric amounts with heme inhibits oxidative modification of LDL and subsequent EC damage [91]. This particular interaction with LDL could participate in the development of atherosclerosis [84] through either hemorrhagic processes in atherosclerotic plaques or in cases of persistent intravascular hemolysis. Premature atherosclerosis characterized by arterial stiffness has been reported in children and young adults with major b-thalassemia [92,93].

#### 4.1.3. Ferroptosis

Ferroptosis is a regulated, cell-death pathway characterized by an iron-dependent decrease in glutathione (GSH) levels and the accumulation of lipid hydroperoxides to lethal levels [94,95]. Numerous genes have been implicated in the regulation of ferroptosis, among them the NRF2 transcription factor that promotes resistance to this process, especially in upregulating the expression of key proteins in iron signaling (i.e., ferritin, ferroportin), or else the glutathione synthesis pathway [96]. A normal physiological function for ferroptosis has not yet been established [95]. Conversely, its role in various pathologic conditions is increasingly suggested, in particular neurological pathologies (i.e., neurodegenerative diseases, stroke, intracerebral hemorrhage, traumatic brain injury) [97] and tubular kidney disorders (i.e., ischemia-reperfusion injury, kidney degeneration) [98]. Its direct impact on the endothelium remains poorly studied, probably because ECs are not a site of preferential iron accumulation (compared with proximal tubular cells, for example). Ferroptosis has been proposed as a mechanism of endothelial toxicity mediated by PM2.5 particulates. In vitro, the internalization of PM2.5 into endothelial cells altered their viability in association with an increase in cellular iron content, ROS production and GSH depletion. The PM2.5-mediated production of proinflammatory cytokines decreased in the presence of ferrostatin-1 (Fer-1), a selective inhibitor of ferroptosis [99], or deferoxamine mesylate, an iron chelator [100]. In a cardiac grafted murine model, the treatment of heart recipients with Fer-1 inhibited the death of cardiomyocytes and fibroblasts, but not ECs. However, the endogenous substances released during ferroptotic cell death (probably DAMPs and alarmin) can trigger TLR4 signaling in ECs which promotes neutrophil adhesion to coronary veins in a type I IFN–dependent fashion [101]. Among the others actors of vascular homeostasis, platelets could also be impacted by ferroptosis; in vitro, this process was generated in platelets by 10–25µM heme through the HO-1-mediated release of iron and the subsequent generation of hydroxyl radicals [102]. 

Of note, ferroptosis is not the only cell death mechanism mediated by heme. Free heme can mediate neuronal necroptosis directly by IL-1R1-dependent assembly of the necrosome complex, triggering cell death [103]. Moreover, heme causes pulmonary microvascular endothelial barrier dysfunction via necroptotic cell death [104].

### 4.2. Dysregulation of Vasomotor Tone 

NO is a key player in vascular homeostasis. Indeed, it is a powerful inducer of endothelium vasodilation [105,106]. Endothelial NO synthesis is controlled by endothelial NO Synthase (eNOS), a constitutive enzyme that catalyzes oxidation of L-Arginine to NO and L-citrulline in the presence of NADPH (nicotinamide adenine dinucleotide phosphate). Thereafter, NO diffuses across the membrane into smooth muscle cells to induce cell relaxation by increasing the intracellular rate of cGMP (cyclic Guanosine MonoPhosphate). 

Intravascular hemolysis induces rapid NO depletion. As stated above, NO is consumed in reactions with oxyHb(Fe^2+^) to generate MetHb(Fe^3+^). In addition, hemolysis can inhibit its synthesis in releasing erythrocyte arginases, which convert L-arginine (necessary for NO synthesis) into ornithine. Capturing NO produced by ECs, oxyHb inhibits its paracrine diffusion into vascular smooth muscle cells and alters vasomotricity [28,107]. Many studies have shown this alteration of vascular tonus resulted in systemic arterial or pulmonary hypertension, as well as a modification of organ perfusion [108,109,110]. Inhibition of the NO/Hb interaction, in a hemoglobin mutagenesis rat model, has shown decreased vasoconstriction and arterial hypertension after infusion of free hemoglobin [111]. Moreover, due to its low molecular weight (32 kDa), the dimeric form of Hb can diffuse through the endothelium via sub-endothelial and peri-vascular spaces, where it may scavenge sub-endothelial NO [112]. Several studies have shown a decrease of the vasoactive effect of free Hb upon polymerization due to an increased molecular weight [113]. For instance, one of the inhibitory effects mediated by Hp is the formation of high molecular weight complexes (>150 kDa) which remain in blood vessels [114]. Another mechanism of vasoconstriction involves heme. It was found to increase portal vein pressure in an ex vivo model of rat livers perfusion via a mechanism that involves hepatic stellate cell-mediated sinusoidal constriction [115]. Such sinusoidal constriction is a hallmark of microcirculatory failure under stress conditions. 

Recently, it was shown that cell-free Hb from cerebrospinal fluid in vascular structures is the primary cause of aneurysmal subarachnoid hemorrhage pathophysiology [116]. Indeed, RBC destruction in the subarachnoid space leads to an accumulation of cell-free Hb, exceeding haptoglobin scavenger capacity in the cerebrospinal fluid. This unbound Hb thus promotes spasms in cerebral arteries and smaller parenchymal arterioles [116]. In a sheep model, administration of Hp into the cerebrospinal fluid inhibited Hb-induced cerebral vasospasm and preserved vascular NO-signaling. 

### 4.3. Heme as a Damage-Associated Molecular Pattern (DAMP)

Heme acts as an alarmin and a prototypic DAMP that is recognized by pattern recognition receptors, mediating inflammatory processes by targeting innate immune cells and ECs [117,118].

#### 4.3.1. Binding to Toll-Like Receptor 4 (TLR4)

The first evidence that TLR4 could serve as a sensor for extracellular heme came from *in vitro* studies of macrophages, where the heme-induced TNF-α production was inhibited by a TLR4-specific inhibitor TAK-242 [119]. Heme was also shown to be capable of polarizing macrophages by promoting phenotype-switching to type 1 (M1), and even switching from type 2 (M2) to type 1 (M1) through TLR4 in vitro [120]. A TLR4-mediated response was detected using protein-free heme on macrophages, but was lost when heme was complexed with albumin [65]. 

A series of in vitro experiments showed that heme is a potent activator of ECs, in part triggered by TLR4 [79,121]. This is a rapid phenomenon, occurring in less than 30 min, and results in signal transduction, mobilization of the endothelial Weibel Palade bodies and NF-kB activation, thus conferring a pro-inflammatory and pro-thrombotic phenotype to the ECs (HUVEC used as a model in both studies [79,121]). The endothelial activation in the liver was also attenuated in TLR4^-/-^ mice in a murine model of intravascular hemolysis [121].

#### 4.3.2. Binding to G Protein-Coupled Receptors (GPCRs)

Exposure to heme induces neutrophil extracellular trap formation both in vitro and in vivo, which was inhibited by the use of Hx [122,123]. Interestingly, this terminal-activated neutrophil state was ROS-dependent, since anti-oxidants abrogated heme-induced extracellular neutrophil trap release. Interestingly, an implication of G protein-coupled receptors (GPCRs) in heme-mediated neutrophil activation has been suggested, using in vitro and in vivo experimental settings [124,125]. Indeed, pertussis toxin, an inhibitor of Gαi/o GPCRs, blocked neutrophil migration after stimulation with heme [125]. In addition, ROS production by neutrophils was determined to be Gαi and PKC dependent [125]. Unfortunately, no recent study has taken this work further and there remains a lack of evidence for the implication of GPCRs in the heme sensing of ECs, while the GPCR(s) involved remain unidentified. 

#### 4.3.3. Activation of the Inflammasome

Heme was found to activate NLRP3 inflammasome in LPS-primed macrophages, resulting in IL-1β processing in a spleen tyrosine kinase-, NADPH oxidase-2-, mitochondrial reactive oxygen species-, and K(+) efflux-dependent manner [126]. Heme also stimulated the production of S100A8 by macrophages, which acted as an autocrine priming signal for the macrophages’ heme-induced pro-inflammatory responses (for example NLRP3 inflammasome-mediated IL-1β secretion) in a hemolytic inflammation mouse model, as well as in vitro [127]. 

On ECs (HUVEC), heme upregulated NLRP3 expression and induced active IL-1β production, processes which were greatly amplified by LPS priming [128]. Moreover, heme administration in mice induced caspase-1 activation and cleavage of IL-1β, which was not observed in NLRP3^-/-^ animals. This process contributes to the inflammation triggered in the hemolysis context. 

### 4.4. Heme as an Activator of Complement Alternative Pathway 

**Figure 4:**
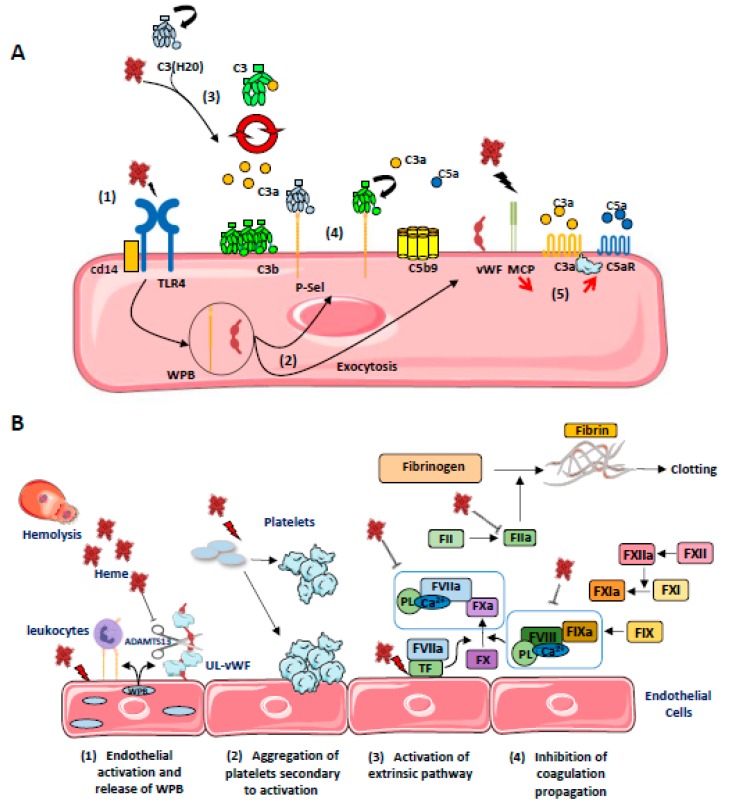
(**A**) Schematic representation of the different (non-exclusive) mechanisms leading to the activation of complement on ECs by heme: (1) Heme released from RBC activates TLR4 on ECs which promotes (2) a rapid exocytosis of WPB, leading to the release of vWF and P-Sel expression; (3) Heme induces C3(H20) generation and promotes the formation of alternative C3 convertases in fluid phase, which cleaves native C3; (4) C3b can be anchored covalently on cell membrane and also, together with C3(H20), non covalently on P-selectin allowing local formation of C3 convertases, release of anaphylatoxins C3a and C5a and C5b9 formation; (5) Heme also decreases MCP expression on ECs while it promotes C3aR and C5aR expression, increasing EC activation. (**B**) Schematic representation of the different (non-exclusive) mechanisms leading to hemostasis activation on ECs by heme. (1) Free heme activates ECs leading to the release of P-selectin and ultra-large von Willebrandt Factor (UL-vWF) from WPB. P-selectin recruits leukocytes by interacting with PSGL-1 (P-selectin glycoprotein ligand 1). (2) Platelet activation and aggregation is mediated by different mechanisms: free heme inhibits the cleavage of UL-vWF in vWF by ADAMTS13. Excess prothrombotic UL-vWF interacts with GPIb (Glycoprotein Ib) on platelets and induce their activation and aggregation. (3) Free heme upregulates the expression of tissue factor (TF) on ECs, activating the extrinsic pathway of coagulation. (4) Paradoxically, heme is also an inhibitor of the coagulation system through co-factors—FV and FVII—inhibition as well as thrombin inhibition.

Heme, but not Hb, is a potent modulator of the innate immune complement system [9] (Figure 4A). It inhibits the target molecules’ recognition by the classical complement pathway initiating protein C1q (Roumenina et al. 2011), but activates the alternative complement pathway [121,129,130,131,132]. The first evidence for heme-mediated complement activation came in 1978, when Wilson et al. showed in vitro that the addition of hemoglobin solutions (prepared from erythrocyte lysates from healthy or sickle cell patients) to human serum triggered activation of the alternative complement pathway, revealed by the presence of C3 and persistent FB cleavage products. At that time, the possible role of complement in the genesis of the prothrombotic endothelial phenotype observed in acute intravascular hemolysis was suggested. These results were questioned, given the use of potentially contaminated hemoglobin solutions under experimental conditions. However, it has since been shown that hemoglobin enhances the ability of bacterial endotoxins to activate coagulation [133] and it has been further suggested that the same may be true for complement activation [134]. 

These early observations were explained by Pawluczkowycz et al. in 2007, who described an activation of the alternating pathway in serum under the effect of heme [129]. This activation was accompanied by the deposition of C3 fragments (C3b, iC3b, C3dg) on the surface of erythrocytes, in part via the CR1 receptor, and promoting their clearance by the phagocytic system. They therefore reported a new mechanism to explain the amplification of hemolysis by free hemoglobin and its derivatives in the context of malaria. 

Our team subsequently confirmed the activation of the alternate pathway by heme, finding an increase in C3a, C5a, and sC5b-9 in normal human serum exposed to heme. This alternate pathway activation was demonstrated on ECs exposed to heme with an increase in C3b and C5b-9 deposition on the cell surface. Complement deposits were exacerbated in the presence of serum from patients with atypical hemolytic uremic syndrome possessed of a deregulated complement due to genetic abnormalities [135,136]. Heme would interact with the C3 molecule, near the thioester bridge, inducing C3-C3 complexes and promoting the formation of C3 and C5 hyperactive convertases [130]. Moreover, upon brief exposure to heme (30 min), ECs (HUVEC, HMEC, and glomerular ECs) exhibited decreased surface levels of complement regulators MCP (CD46) and DAF (CD55), thus reducing their capacity to control the activation of complement at their surface [121,130,132,137]. In addition, heme promotes rapid exocytosis of Weibel–Palade bodies (in less than 30 minutes), leading to membrane expression of P-selectin and the release of prothrombotic vWF factor at the cell surface [79,130], P-selectin serves as an anchoring platform for C3b and C3(H_2_O), amplifying the alternative pathway at the ECs’ surface [121,138,139]. RBC microvesicles from SCD patients activated the complement in a partially heme-dependent manner in serum and on ECs in vitro [132]. These results therefore strongly suggested that heme could amplify endothelial and thrombosis lesions in complement-mediated TMA, as well as endothelial lesions and vaso-occlusion in SCD. 

### 4.5. Coagulation and Thromboinflammation

Thrombotic complications frequently affect the progression of some hemolytic diseases such as sickle cell disease [140], thalassemia [141,142], or paroxysmal nocturnal hemoglobinuria [143]. In addition, an increase in prothrombin and thrombin times has been reported after intravenous injection of hematin in healthy volunteers, and associated with the occurrence of thrombophlebitis in 45% of cases (4/9 patients) [144]. Similarly, thrombotic complications have also been described in patients with intermittent acute porphyria treated with hematin infusion [145,146]. The different (non-exclusive) mechanisms leading to hemostasis activation on ECs by heme are depicted at Figure 4B.

In a whole blood model, heme induced complement activation and a thromboinflammation response, as measured by upregulation of IL-6, IL-8, TNFa, MCP-1, MIP-1α, IFN-γ, LTB-4, MMP-8 and MMP-9, and IL-1Ra, upregulation of CD11b on granulocytes and monocytes, monocytic tissue factor expression, and prothrombin cleavage [131]. These could be controlled at best by a combination of complement C5 blockade and inhibition of the TLR-associated CD14. 

On ECs, heme induces retraction as well as promoting adhesion of platelets to bovine aortic ECs (BAEC) in vitro [147]. This could be related to the mobilization of the Weibel–Palade bodies, resulting in surface expression of P-selectin and release of ultra-large VWF multimers [79,130]. Heme induces endothelial tissue factor expression [148,149]; it activates platelets, but inhibits thrombin and plasmin; heme also binds to factor VIII and inhibits its interaction with activated coagulation factor IX [150]. Taken together, then, the data in the literature suggest that the release of heme initiates the extrinsic coagulation pathway via the upregulation of tissue factor in ECs and leukocytes, but subsequently blocks the propagation of coagulation by inhibiting FVIII and FV, and by inhibiting the conversion of fibrinogen into fibrin and hence the formation of fibrin clots. It has been suggested that the delicate balance between the anti-coagulant and pro-thrombotic effects of heme depend upon the availability of heme to interact with different proteins and cell-surface receptors [9]. The overall effects of heme on homeostasis might well be potentiated by additional factors, such as ongoing inflammation.

## 5. Heme as a Cell-Protective Agent

The father of toxicology, Paracelsus, concluded in the 16th century that “Sola dosis facit venenum” or “The dose makes the poison”. This notion is reflected by the concept of “hormesis”, which is any process in a cell or organism that exhibits a biphasic response upon exposure to increasing amounts of a substance or condition [151]. This implies that there is a favorable biological response during low exposures to toxins and other stressors. Heme falls into this category of substances. It induces the upregulation of HO-1, the enzyme that degrades it to anti-oxidant and cytoprotective metabolites. 

In multiple models of transplantation, sepsis or ischemia-reperfusion injury, pre-treatment of the experimental animals with low doses of heme attenuates inflammation, oxidative stress, cell activation, infiltrates of neutrophils and macrophages, and tissue rejection or injury [60,152,153,154]. All these effects are attributed to an upregulation of HO-1 prior to the insult. Therefore, different cell types which overexpress HO-1, such as macrophages, ECs, etc., are readily protected and can adapt to the stress condition, which is often accompanied by tissue destruction and heme overload. Since cobalt protoporphyrin (an analogue of heme where cobalt replaces iron) is a much stronger inducer of HO-1, it is more often used in a pre-treatment regime in experimental models [9,118]. Zinc protoporphyrin, on the contrary, induces HO-1 expression but blocks it activity and is used as a negative control in model conditions. 

Both heme and heme arginate (a therapeutic agent for porphyria) induce HO-1 expression in healthy volunteers [155,156] and heme arginate ameliorates experimental ischemia-reperfusion injury in healthy individuals [157]. These data generated significant hope for the therapeutic application of HO-1 induction. However, in a study of patients with metabolic syndrome heme arginate did not improve endothelial function or insulin sensitivity, but significantly reduced the vasodilator response to nitroglycerine [158]. These negative findings contrast with the preclinical data, perhaps due to short duration of therapy and limited HO-1 induction, as well as interference by markedly elevated plasma heme levels [158]. Future studies should pay attention to the delicate balance between sufficient dosage and timely normalization of plasma heme levels.

## 6. Intravascular Hemolysis as a Second Hit in Renal Vascular Disorders 

An overview of the literature reveals several aspects of heme biology: (i) it has a dualistic nature being an inducer of inflammation and tissue damage as well as cytoprotective via HO-1; (ii) it is difficult to quantify it in vivo; and (iii) its toxic properties are strongly amplified in presence of additional stimuli, such as infection, inflammation, or the presence of mutations for example in Hb or complement genes which occur, respectively, in SCD and aHUS. These stress factors were shown to synergize with heme to promote EC activation and tissue injury. This growing body of data supports the two-hit (or multiple hit) model, in which heme may not induce clinically relevant endothelial damage alone but amplifies the effects of a primary hit and/or a pre-existing genetic abnormality. In the next section we discuss this two-hit model using the example of atypical HUS, a hemolytic disease targeting glomerular ECs. 

### 6.1. Focus on Atypical HUS 

#### 6.1.1. A Multiple Hit Disease

Atypical HUS (aHUS) is a model of disease mediated by complement dysregulation [159]. Indeed, genetic variants of complement-related proteins such as FH, FI, MCP, FB, C3 were identified in more than 50% of cases, but these abnormalities are generally not sufficient to trigger the disease. Clinical studies have shown sporadic forms to represent about 85% of cases, despite familial inheritance of the mutation, suggesting that while genetic background is not a cause it nevertheless predisposes for the disease [160]. In a study identifying 27 patients carrying combined mutations among 795 aHUS-patients, only 8 to 10% of patients carrying FH, FB, or C3 mutation had a second mutation against 25% of patients with an MCP or FI mutation. This could suggest a different level of risk factor according to the altered protein; higher for the FH, C3 than MCP or FI. In addition, Bresin et al. reported in this work an average penetrance of 10% in cases of isolated mutation, against 55% in presence of two mutations and 100% when three mutations coexisted (2/2 patients). Some polymorphisms, particularly MCP_ggaac_ and FH_gtgt_ haplotypes, also increased penetrance and severity of aHUS episode [161]. Besides genetic risk factors, additional environmental factors are generally necessary to result in the overwhelming of complement regulation capacities. Therefore, a variety of events have been associated with aHUS including infections, pregnancy, transplants, drugs, autoimmune conditions, and metabolic conditions [162]. 

Histologically, this rare disease is due to the development in microvessels (vessel diameter < 200 µm) of TMA lesions, characterized by a swelling of sub-endothelial space owing to the accumulation of flocculent material and leading to EC detachment in the lumen of microvessels. Fibrin-platelet thrombi formation, subsequent to exposure of the glomerular basement membrane, leads to occlusion of glomerular capillaries. This occlusion may promote RBC destruction on the platelet-forming clot, thus leading to a local hemolysis, enhancing the stress of the endothelium and leading to the typical clinical presentation associating mechanical hemolysis, thrombocytopenia, and organ ischemia. Interestingly, although the complement overactivation is systemic, a particular renal tropism of the TMA lesions exists. Indeed, extra-renal complication occurs only in 8–25% in adult patients [163]. 

#### 6.1.2. Why Kidneys? 

**Figure 5:**
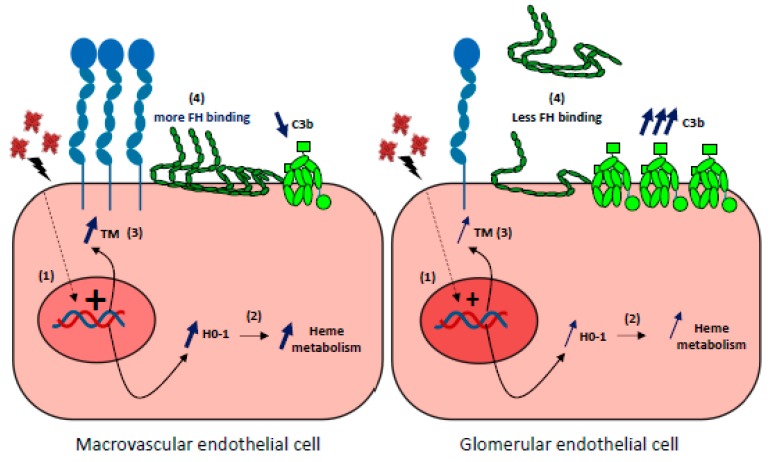
Glomerular endothelial cells susceptibility to hemolysis (**1**) Upon hemolysis, glomerular endothelial cells display a weaker capacity than macrovascular or other microvascular EC to upregulate the expression of HO-1 and thrombomodulin (TM) genes. (**2**) Subsequent relative deficiency in HO-1 will result in a stronger heme-mediated complement activation on the endothelium. (**3**) Weaker expression of TM will aggravate the pro-coagulant phenotype of ECs and increase complement activation as TM is a negative regulator of the complement system. (**4**) FH binding to heme-exposed, glomerular ECs is decreased compared with macrovascular Ecs, and will result in a more important complement activation on ECs.

This susceptibility of renal ECs to complement overactivation remains incompletely understood. No major differences exist in the basal expression of complement regulators or C3 deposits in resting-state differences between glomerular and other ECs [3,164]. High expression of complement proteins in kidneys could explain the greater susceptibility of glomerular ECs to complement attack. The EC-produced C3 and FB are, however, lower compared with the liver-derived C3 and FB in blood, to which endothelium is exposed in vivo. Hypotheses involving for example the specific rheology of kidney or the features of glycocalyx have been proposed. In addition, it has recently been demonstrated that glomerular ECs do not adapt to the stress imposed by hemolysis owing to inefficient HO-1 upregulation, which amplifies complement overactivation and thrombotic microangiopathic lesions [137]. Indeed, after prolonged exposure to acute hemolysis, higher C3 deposits were found on glomerular ECs when compared with other ECs both in culture and in organs from mice (liver, skin, brain, lungs, and heart). This could be explained by a reduced complement regulation capacity resulting from weaker binding of Factor H and inefficient upregulation of thrombomodulin (TM). Microvascular ECs also failed to upregulate HO-1, normally induced by hemolysis products. Only HUVEC developed an adaptation to heme, which was reversed by inhibition of HO-1 activity. Microvascular ECs, and especially glomerular ECs, thus failed to adapt to the stress imposed by hemolysis and acquired a pro-coagulant and complement-activating phenotype [137]. Together, these findings indicate that the relative vulnerability of glomerular ECs to hemolysis may be a key factor, amplifying complement overactivation and thrombotic microangiopathic lesions (Figure 5).

### 6.2. Particular Kidney Susceptibility to Hemolysis 

#### 6.2.1. Traditional View: Predominance of Tubular Suffering 

Renal susceptibility to hemolysis has long been studied in animal models of experimental, acute renal failure following infusion of hemoglobin or methemoglobin solutions [165,166]. This vulnerability appears unsurprising, kidneys being the main site of clearance and degradation of free hemoglobin. Indeed, glomeruli can filter dimeric Hb which is then internalized into epithelial cells of the proximal tubules via two apical, pole-located receptors cubilin and megaline [167]. Internalized Hb is dissociated and heme is metabolized in renal proximal tubular epithelial cells by HO-1,which is highly and rapidly expressed in response to oxidative stress events [168]. Again supporting the specific impact of derived-hemolysis products, tubular lesions were exacerbated when heme detoxification systems, such as hemopexin [169] or HO-1 [170], were invalidated.

Tubular damage thus appears to predominate in hemolysis-induced kidney injury. Histological analyses in cases of acute, hemolysis-mediated renal failure classically report proximal tubular hemosiderosis, intra-tubular Hb precipitate and acute tubular necrosis lesions, while the other segments of the nephron, when described, remain subnormal [171]. Among the main mechanisms of hemolysis-induced kidney injury, tubular obstruction by intra-tubular precipitation of hemoglobin and direct heme-toxicity on tubular ECs are prominent, contributing to their apoptosis and detachment. These tubular aspects had been exhaustively described [98,172]. 

#### 6.2.2. Underestimated Renal Vascular Damage in Hemolytic Diseases? 

Notable among the mechanisms of hemolysis-related renal toxicity is NO depletion, responsible for vasoconstriction and decreased renal blood flow and even renal hypo-perfusion. For example, Minneci et al. [108] showed, in a canine model of free water-induced intravascular hemolysis, that cell-free Hb severely reduced NO bioavailability, producing dose-dependent systemic vasoconstriction and impaired renal function. Interestingly, inhaled NO gas (80 ppm) oxidized 85% of the plasma oxyhemoglobin to methemoglobin, thereby inhibiting endogenous NO scavenging by cell-free Hb. As a result, the vasoconstriction caused by acute hemolysis was attenuated and responsiveness to systemically-infused NO donors restored. In the same model, the authors noted an increase in mean arterial blood pressure following NO depletion. Indeed, NO is known to promote natriuresis and diuresis, as well as contributing to adaptation to variations in dietary salt intake in order to maintain normal blood pressure [173]. Therefore, NO depletion could increase kidney damage by promoting systemic hypertension, in addition to renal ischemia [174]. 

Apart from these hemodynamic effects, and despite the obvious endothelial toxicity of free hemoglobin and its derivatives, there is a lack of data about their impact on microvessels, especially glomerular capillaries. In animal models of induced-hemolysis (Hb or hematin infusions, as well as failed detoxification systems), microvascular lesions have not been studied. There are sound arguments for a hemolysis-induced vulnerability of glomerular endothelial cells, however. Firstly, mice lacking functional HO-1 develop a glomerular disease with membranous proliferation, lobularity, crescent formation, and glomerulosclerosis in addition to iron accumulation [175]. The staining used in this study (hematoxylin and eosin), combined with the already severe sclerosis in the 75-week-old HO-1^-/-^ mice, prevented a detailed analysis of glomerular capillaries. In accordance with a ‘glomerular’ susceptibility in HO-1 deficient animals, there have been two case reports of HO-1 deficiency in humans. Both presented hematuria and proteinuria and kidney biopsies revealed a mild mesangial proliferation and thickening of the capillary loop using light microscopy, while electron microscopy revealed endothelial swelling and detachment in the glomerular capillary [55,176,177].

Glomerular involvement in SCD nephropathy also raises the question of a peculiar susceptibility of glomerular ECs to hemolysis injury, especially since albuminuria was shown to be associated with hemolysis markers and improved with hydroxyurea treatment [178]. Interestingly, Merle et al. observed significant C3 deposits in glomeruli in of human SCD nephropathy biopsies and in kidneys of two mouse models of SCD [132]. These complement deposits were reproduced in a murine hemolysis model, via the injection of phenylhydrazine (PHZ), and reduced by heme-scavenging through the administration of hemopexin. Thus, heme-induced complement activation could play a key role in glomerular susceptibility. 

### 6.3. Vulnerability of Glomerular ECs to Hemolysis: Different Therapeutic Strategies

**Figure 6:**
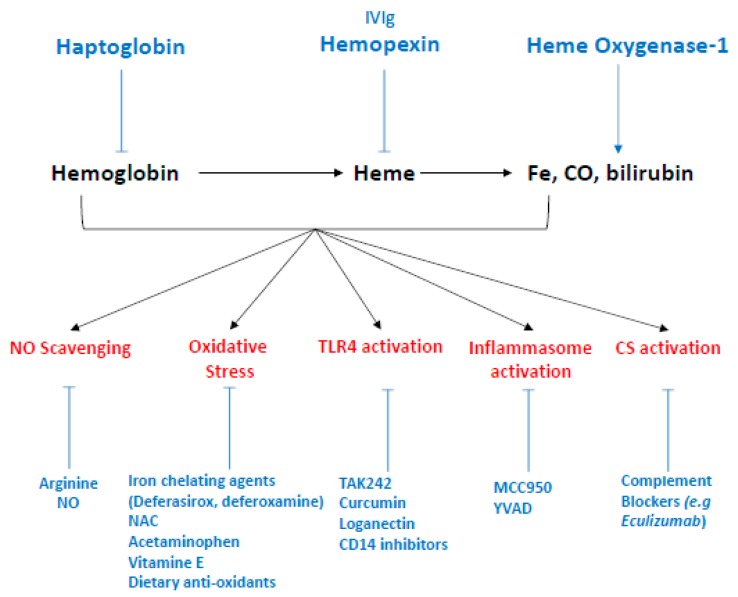
Therapeutic strategies to targeting hemolysis derived products. The antioxidant scavenger proteins haptoglobin and hemopexin bind and neutralize extracellular Hb and free heme in plasma, respectively, and represent the two most promising therapeutic strategies. To a lesser extent, intravenous polyclonal immunoglobulin have been shown to scavenge heme in vitro. Heme-Oxygenase 1 is the inducible isoform of HOs, which enzymatically degrades intracellular heme and produces iron, carbon monoxyde and biliverdine. Potential therapies including gene therapy and HO-1 inductors (e.g., statins) which could upregulate HO-1 expression. More specific interventions could target each side effect induced by hemolysis-derived products such as NO scavenging, counteracted by administration of L-arginine or NO inhalation. Oxidative stress, reduced by acetaminophen, as well as iron chelating agents (deferasirox, deferoxamine) alone or in association with N-acetyl-cysteine (NAC). Vitamin E and dietary antioxidant are also studied in that end. TLR4, the main receptor of free heme, with pharmacological inhibitors—such as TAK 242, curcumin, or loganectin—which directly inhibit TLR4. Likewise, inhibitors targeting its co-receptor CD14 are also worth studying. Inflammasome activation by heme can be specifically reduced by MCC950, a specific inhibitor of NLRP3 or YVAD, themselves specific inhibitors of caspase-1. Finally, heme is an activator of the complement system through the alternative pathway. This activation is blocked by complement inhibitors such as eculizumab, a monoclonal antibody directed against C5.

To stop hemolysis and/or their deleterious effects thus appears to be a pertinent therapeutic target motivating many researchers and giving rise to various treatment suggestions (Figure 6). These therapeutic propositions have recently been detailed elsewhere [98] and we will more particularly discuss the potential for targeting pathways concerning the renal endothelium.

#### 6.3.1. Limiting Complement Activation 

As noted earlier (Section 4.4) and in the preceding section, free heme is an activator of the complement alternative pathway and may explain the presence of complement deposits seen in glomeruli of SCD patients, as well as complement activation amplification seen in aHUS. Therefore, the use of complement blockers should improve glomerular damage in hemolytic diseases. Eculizumab, a monoclonal antibody directed against C5 and the first complement blocker to be approved by the FDA for treatment of paroxysmal nocturnal hemoglobinuria [19] and aHUS [179,180], drastically improved hemolysis and renal survival in those patients. Eculizumab has also shown its efficacy in several case reports of delayed hemolytic transfusion reactions (DHTR) in SCD patients [181] and may prove to be an interesting therapeutic approach in these indications. Complement inhibitors are of great interest to pharmaceutical companies and numerous inhibitors should be released shortly [182]. 

#### 6.3.2. Blocking Hemolysis-Derived Products

Another approach could consist in optimizing the detoxification systems in order to limit the circulating levels of free Hb and heme. Several research teams and pharmaceutical companies are now focusing on the development of Hp treatments and hemoglobin or heme scavengers. Molecular approaches have shown that Hp treatment in hemolytic mice models prevents a molecular stress signature in the kidney [183]. For instance, increased expression of HO-1, NGAL, and KIM-1 by injection of stored RBC was prevented by the addition of Hp (but not by Hx). In addition, Hp (but again, not Hx) was able to prevent NO depletion by Hb in vitro and ex vivo, and thus prevented Hb-induced systemic hypertension by compartmentalizing cell-free Hb in plasma [74,184]. Similar results were observed in a guinea pig model exposed to transfusions of senile erythrocytes: in the absence of treatment with purified haptoglobin, the animals rapidly developed (24 h) a combination of intravascular hemolysis, hypertension, vascular lesions (necrosis/coagulation), hemoglobinuria, and acute renal failure [185]. Purified haptoglobin has been available in Japan since 1985 for the treatment of hemolysis due to extracorporeal circulation, burn injuries, and trauma with massive transfusions. Subsequent findings from several small clinical studies or “case report” suggest the value of this treatment in the prevention of acute renal failure [114], particularly in cardiac surgery for extracorporeal circulation [186]. Nevertheless, the use of purified haptoglobin is not widespread, perhaps because of production difficulties or insufficient efficacy, and other therapeutic targets are needed.

Hx is the natural scavenger of heme and is currently being actively investigated. Indeed, Hx was shown to protect against heme toxicity in hemolytic disorders such as SCD and malaria [79,187]. Vinchi et al. showed, in an in vitro model of endothelial cells (HUVEC) exposed to hematin as well as in two mouse models of β-thalassemia and SCD, that the addition of hemopexin was associated with a decrease in endothelial expression of adhesion molecules (VCAM-1, ICAM-1, E-selectin), a decrease in ROS production, and an increase in NO bioavailability [29]. Therefore, we anticipate a beneficial effect of hemopexin on glomerular damage, especially since Merle et al. demonstrated that Hx decreased P-selectin expression and counteracted heme-induced complement deposition on glomerular ECs [121]. There is currently no clinical experience with hemopexin, but the beneficial effects observed in vivo should be translatable to the clinic.

#### 6.3.3. Stimulating the Expression of HO-1 

It has been proposed that the beneficial effects of Hp and Hx against vaso-occlusive crisis (VOC) and inflammation in Townes-SS mice could be due to increased HO-1 activity independent of Hb and heme scavenging. Likewise, Belcher et al. have developed the use, in a mouse sickle cell model, of a targeted *hmox-1* gene therapy using transposon which led to an increase in hepatic HO-1 expression. They showed, in mice exposed to hypoxia/reoxygenation, a decrease in certain proinflammatory signaling pathways (MAPK, NF-κB), in the expression of adhesion molecules (VCAM-1) and in vascular stasis in subcutaneous veins [188], but did not study kidney function nor glomerular damage. As discussed earlier, glomerular ECs are more susceptible to hemolysis-driven injury partly because they fail to upregulate HO-1. Thus, therapeutically targeting HO-1 could be of great interest to restore glomerular insufficiency. To our knowledge, no such therapy has been developed to that end, but several already-approved molecules are known to be HO-1 inducers. For example, Heme arginate is approved in Europe for treatment of hepatic porphyrias and was found to upregulate HO-1 in patients receiving deceased-donor renal transplants [189]. Likewise, statins [190,191] and 5-aminosalicylic acid [192] both lead to an upregulation of HO-1 and could be investigated in the context of hemolytic diseases. Indeed, a better understanding of HO-1 downstream signalization has led to several novel candidates for targeted HO-1 upregulation which are reviewed elsewhere [193], but it should be remembered that this therapeutic strategy is controversial, especially in some degenerative brain disorders [61].

#### 6.3.4. Others Potential Targets? 

Others treatments such as TLR4 inhibitors (Figure 6) aim to decrease oxidative stress, inflammasome activity and neutrophil infiltration. Increasing NO bioavailability would more specifically target the vascular effects of hemolysis and could restore renal blood flow and limit glomerular damage, as well as prevent the development of arterial hypertension. Kaul et al. showed, in a mouse sickle cell model (BERK), that arginine administration decreased hemolysis and oxidative stress and increased the bioavailability of NO [194]; in another mouse model of sickle cell disease (Hbb^s^/ Hbb^s^ SAD mice), a beneficial effect on pulmonary vascular vasoconstriction and bronchial inflammation was observed under the effect of inhaled NO [195]. Likewise, Hydroxyurea, the only therapeutic alternative commercially available for SCD patients, acts by promoting fetal Hb levels but also by increasing NO production, and has been shown to reduce albuminuria and protect renal function. This was strongly associated with decreases in levels of hemolysis markers [178]. It has been shown, in patients undergoing multiple valve replacement and prolonged cardiopulmonary bypass, that administration of inhaled NO decreased the incidence of AKI, as well as major adverse kidney events, up to one year after surgery [196]. 

## 7. Conclusions

Endothelium is a complex tissue with various specificities dependent upon the vascular tissue, and dynamically regulated by numerous systems including immunity, coagulation and vascular tone (for example). It is therefore a central element of homeostasis, but also of many diseases—both as an actor and a target of the pathological process. Our review has focused on the deleterious endothelial effects of intravascular hemolysis and its by-products, in particular heme, in order to illustrate the multiple-hits concept of vascular diseases. Typically, in our example, an initial aggressive event leads to the lysis of red blood cells and the release of molecules which are highly toxic for the endothelium. 

This multiple-hits concept has also been proposed in other vascular diseases affecting various tissues. In vitro, the exposure of HUVECs to heterodimeric S100A8/100A9 was reportedly only associated with an increased protein expression of inflammation markers (VCAM-1, ICAM-1, IL6) in cases of pre-treatment with an AGE-albumin solution [197]. This could indicate a synergistic effect for these two ligands of the receptor of advanced glycation end products (RAGE) which drives significant endothelial activation, and the authors suggest a potential role for triggering atherosclerosis, particularly in patients with diabetes mellitus and/or chronic kidney disease [197]. Similarly, we have reported that exposure of resting ECs (both HUVEC and glomerular ECs) to dysregulated-complement sera (sera from aHUS patients, FH-depleted serum, or a blocking FH with an inhibitory antibody) did not lead to complement membrane deposition (except in one case) or fluid phase activation, while these consequences were observed if cells were previously incubated with pro-inflammatory cytokines [135]. The pathogenesis of antiphospholipid syndrome, a heterogeneous vascular disease characterized by the occurrence of thrombotic and/or obstetrical events in various vascular tissues, requires antiphospholipid antibodies among which anti-β2 glycoprotein I antibodies (GP1) provide the main pathogenic subset. These antibodies are not sufficient in themselves to drive thrombotic events, however, and Borghi et al. proposed a two-stage model in which LPS provides the second hit by upregulating the endothelial expression of its natural receptor TLR4. This leads to increased GP1 expression in vascular tissues and the TLR4-mediated, anti-GPI-induced intracellular signaling cascade [198,199]. A two-hit model is also proposed in hereditary pulmonary arterial hypertension (hPAH), in which *Bmpr2* mutations is a strong susceptibility factor but where only 20% of carriers develop the disease. Pulmonary inflammation, generated by overexpressing 5-lipoxygenase (a condition observed in hPAH patients), was associated with severe PAH in Bmpr2^+/−^ rats, while neither the single ‘hit’ of *Bmpr2* haploinsufficiency nor 5-LO-specific pulmonary inflammation alone initiated the disease—their combined presence was required to develop a lethal PAH [200]. 

Such examples go to illustrate how much the expression of many vascular diseases requires the addition of a risk terrain (mutations, polymorphisms), a favorable environment (circulating autoantibodies, pregnancy), and a triggering factor to unbalance homeostasis. Dissecting the combinations of factors involved in any given disease will allow us to improve their clinical management and hence patients’ prognoses.

## Figures and Tables

**Table 1:** Main hemolytic diseases.

Extravascular Hemolysis		*Main Mechanisms*
**Intrinsic RBC defects**		
Hemoglobinopathies	e.g., SCD, thalassemia, unstable Hb disease	A normal 7µ RBC can deform itself and pass through the 3µ openings in the splenic cords. RBCs with structural alterations of the membrane surface are unable to traverse this network and are phagocytosed by macrophages.
RBC enzyme deficiencies	e.g., G6PD, pyruvate kinase deficiencies	
RBC membrane disorders	- Constitutional (e.g., spherocytosis, elliptocytosis)	
	- Acquired (e.g., PNH)	
**Extrinsic RBC defects**		
Binding to Ig	- Autoimmune hemolytic anemia (e.g., warm- or cold reacting antibodies); systemic diseases (e.g., lupus erythematosus, sclerodermia);	Cause of structural alterations: - Poor deformability (change of hydration, cytoskeletal integrity, membrane protein phosphorylation, metabolism and the integrity of Hb) [11]; - Modifications of RBC membrane composition (e.g., PS exposure that is as a marker of RBC apoptosis and natural cell senescence [14]; alteration of lipid composition as in hepatic cirrhosis [15]); - Overwhelming oxidative stress or decreased energy production; Membrane coating with IgG and/or opsonins [16].
	- intravenous immune globulin infusion	
Extrinsic agents	- Infections (e.g., bartonella, babesia, malaria)	
	- Oxidant agents (e.g., dapsone, nitrites, aniline dyes)	
	- Other agents (e.g., lead, copper poisoning; snake and spider bites)	
Liver diseases	Hepatic cirrhosis	
Hypersplenism	All causes of splenomegaly	Sequestration
**Intravascular Hemolysis**		***Main Mechanisms***
“Crisis” during extravascular hemolytic disease	Intrinsic and extrinsic RBC defects (cf. above)	Added event: Complement fixation (most often via IgM) and direct lysis Mechanical, shear-force associated RBC lysis; Membrane- or hemoglobin-disorder associated RBC lysis.
Alloimmune hemolytic anemia	Transfusion reactions; hemolytic disease of the newborn; intravenous immune globulin infusion	Membrane coating (Ig, complement)
Osmotic	Freshwater drowning	Osmotic lysis
Mechanicals	TMA, DIC	Fragmentation due to micro thrombi
	malignant hypertension	Fragmentation
	mechanical heart valves, recent bypass surgery,	direct mechanical trauma

AIHA: autoimmune hemolytic anemia; G6PD: glucose-6-phosphate dehydrogenase; DIC: disseminated intravascular coagulation; Ig: immunoglobulin; PNH: paroxysmal nocturnal hemoglobinuria; PS: phosphatidylserine; RBC red blood cells; SCD: sickle cell disease; TMA: thrombotic microangiopathy.

## Abbreviations

ATP	Adenosine 5’ triphosphate
ADP	Adenosine 5’ diphosphate
ALIS	Aggresome-like induced structures
*AIHA*	*Auto-immune hemolytic anemia*
BAEC	Bovine aortic ECs
CO	Carbon monoxide
cGMP	Cyclic Guanosine MonoPhosphate
DAMPs	Damage-associated molecular patterns
DAF	Decay Accelerating Factor
DHTR	Delayed Hemolytic Transfusion Reactions
DIC	Disseminated intravascular coagulation
ECs	Endothelial cells
eNOS	Endothelial NO synthase
G6PD	Glucose-6-phosphate dehydrogenase
GSH	Glutathione
GPCRs	G protein-coupled receptors
Hsp	Heat shock protein
HO	Heme oxygenase
Hb	Hemoglobin
HUS	Hemolytic uremic syndrome
Hx	Hemopexin
HUVEC	Human Umbilical Vein Endothelial Cells
Ig	Immunoglobulin
F	Factor
FH	Factor H
FB	Factor B
LDH	Lactate dehydrogenase
LDL	Low-density lipoprotein
MCP	Membrane cofactor protein
NO	Nitric oxide
PNH	Paroxysmal nocturnal hemoglobinuria
PS	Phosphatidylserine
ROS	Reactive oxygen species
RBC	Red blood cells
RES	Reticuloendothelial system
SCD	Sickle cell disease
TF	Tissue factor
TM	Thrombomodulin
TMA	Thrombotic microangiopathies
TLR	Toll-like receptor
